# Facilitating knowledge transfer: decision support tools in environment and health

**DOI:** 10.1186/1476-069X-11-S1-S17

**Published:** 2012-06-28

**Authors:** Hai-Ying Liu, Alena Bartonova, Panagiotis Neofytou, Aileen Yang, Michael J Kobernus, Emanuele Negrenti, Christos Housiadas

**Affiliations:** 1 NILU - Norwegian Institute for Air Research, Instituttveien 18, 2027 Kjeller, Norway; 2 National Centre for Scientific Research Demokritos, Agia Paraskevi Attikis, P.O. Box 60228, 153 10 Athens, Greece; 3 Italian National Agencies for New Technologies, Energy and the Environment (ENEA), Lungotevere Thaon di Revel, 76, 00196, Roma, Italy

## Abstract

The HENVINET Health and Environment Network aimed to enhance the use of scientific knowledge in environmental health for policy making. One of the goals was to identify and evaluate Decision Support Tools (DST) in current use. Special attention was paid to four “priority” health issues: asthma and allergies, cancer, neurodevelopment disorders, and endocrine disruptors.

We identified a variety of tools that are used for decision making at various levels and by various stakeholders. We developed a common framework for information acquisition about DSTs, translated this to a database structure and collected the information in an online Metadata Base (MDB).

The primary product is an open access web-based MDB currently filled with 67 DSTs, accessible through the HENVINET networking portal http://www.henvinet.eu and http://henvinet.nilu.no. Quality assurance and control of the entries and evaluation of requirements to use the DSTs were also a focus of the work.

The HENVINET DST MDB is an open product that enables the public to get basic information about the DSTs, and to search the DSTs using pre-designed attributes or free text. Registered users are able to 1) review and comment on existing DSTs; 2) evaluate each DST’s functionalities, and 3) add new DSTs, or change the entry for their own DSTs.

Assessment of the available 67 DSTs showed: 1) more than 25% of the DSTs address only one pollution source; 2) 25% of the DSTs address only one environmental stressor; 3) almost 50% of the DSTs are only applied to one disease; 4) 41% of the DSTs can only be applied to one decision making area; 5) 60% of the DSTs’ results are used only by national authority and/or municipality/urban level administration; 6) almost half of the DSTs are used only by environmental professionals and researchers. This indicates that there is a need to develop DSTs covering an increasing number of pollution sources, environmental stressors and health end points, and considering links to other ‘Driving forces-Pressures-State-Exposure-Effects-Actions’ (DPSEEA) elements. Of interest to both researchers and decision makers should be the standardization of the way DSTs are described for easier access to the knowledge, and the identification of coverage gaps.

## Background

Additional knowledge of the complex problems surrounding environment and health (E&H) increasingly highlights questions regarding the relation between policy and research [1]. In environmental risk assessment, Linkov et al [2] illustrate how decision making has moved from an *ad hoc* process towards working within an integrative decision analysis framework. They propose to integrate environmental management within an adaptive management framework, supported by tools, and integrated with methods for management of uncertainty, including the uncertainty of mitigation options. Taking the perspective of environment and health, we have to define a suitable framework, and to find adequate tools that would provide information that is both easy to understand and of sufficient depth to support decision- and policy making.

Decision support tools (DSTs) (also known as decision aids or decision support technologies) permit the making of the decisions based on complex and wide-ranging information. DSTs can take the form of written guidance, data, models and/or software. They aim not only to facilitate decision making, but to help ensure that the process is transparent, documented, reproducible and robust.

The need for decision support is widely recognised. In recent years, a large number of DSTs have been developed, with varying degrees of success in their practical use [3-5]. However, with the growth of DSTs, the resulting advice can be contradictory, as the different DSTs are based on different data sets and models. Thus, information that would permit the evaluation of the DSTs as well as their inputs is important. As a start, an overview of current DSTs is required.

The HENVINET project (Health and Environment Network) had a general aim to create a “permanent network of professionals”. One line of work supporting this aim was to make publicly available information about DSTs providing qualitative or quantitative assessments that underpin decision making in the field of E&H. This could increase the use of DSTs, leading to their better validation, and more discussion on their use.

Decision support tools are undeniably an important mechanism for transfer of knowledge from researchers to decision makers. The goal of the DST Meta database (MDB) is to make available the vast richness of tools to the management process of environmental health. The main objectives of the work included to 1) define a concept of DSTs in E&H fields; 2) identify available DSTs 3) create an open access web-based DSTs MDB; and 4) carry out categorisation, evaluation, validation and application of DSTs. This paper provides an overview of the work undertaken, with the aim to encourage and facilitate additional effort in making DSTs better known, more used, and therefore, more useful.

## Concept of decision support tools

In the broadest sense, a DST is any guidance, procedure, or analysis tool that can be used to help support a decision [6-9]. Within HENVINET, a DST is a tool that supports decision makers to make decisions in the E&H sector, in particular to propose actions and policies for reducing the burden of environmental stressors on human health. HENVINET defines DSTs as: *any tool based on E&H knowledge that can be used in different decision making contexts: from every day operation of health practitioners to strategic long term planning and implementation of policies for reducing the negative effects of environment on health*. Most often, DSTs are in the form of written guidance, or software. Written guidance is frequently provided by regulatory agencies as a means of ensuring a standardized, reproducible approach to reaching a decision. In many cases, this guidance is translated into computer software. Software tools are also developed to assist in the decision process for computationally intensive analysis (e.g., geo-statistical modelling and multi-criteria analysis), and for mapping the spatial relationship between environmental stressor data and physical features such as buildings, roads (e.g. ArcGIS). Software tools are categorized as data-driven or model-driven DSTs depending on the output of the tools [10].

In HENVINET, a reference concept is the World Health Organisation (WHO) full chain DPSEEA (Driving forces-Pressures-State-Exposure-Effects-Actions) approach, which is identified also as one fundamental concept of the EHAP (Environment and Health Action Plan) [11,12]. Therefore we defined an E&H DST to include models and/or data within at least two of the following areas: environmental stressors’ emissions, their transport and dispersion in the environment, pathways to humans, behaviour and exposure of the population, health effects with reference to the four EHAP priority issues: asthma and allergies, cancer, neurodevelopment disorders and endocrine disruptor mediated-diseases.

## Methods

### Database concept

The database has been designed as a system of “attributes”, or descriptors, with either pre-described categories or free text (see Additional file 1). The development of the attributes took more than one year. The DPSEEA framework permits the description of both the different elements (drivers, pressures, status, exposure, effect, action), and their links. We have developed different kinds of categorisation for several of the elements. We have gathered contact information, and quality control and assurance information. Several trial runs and a review of different classification systems helped to define which attributes should have prescribed categories, where to allow free text, and where to combine these types. The resulting database permits the user to find information using a search for pre-defined categories and free text. The system also allows commenting on each DST.

Formal validation of each individual DST is the responsibility of its owner or designer (information about such validation may be provided as part of the DST description). We have designed evaluation criteria regarding user friendliness, the design of the DST in relation to the concept of the “causal chain” of DPSEEA, robustness of the tool, user application history and applicability of the tool, and whether or not information about uncertainty is available as part of the output. This information is included in the database.

### Database functionalities

The database offers the four following functionalities: add information on new tools and edit it, search for information on available DSTs, and provide reviews or comments.

1. Adding DSTs: in order to upload a DST, registration is required. An online guideline is provided for new users.

2. Editing existing DSTs: only the provider of the given DST is allowed to edit the already uploaded information.

3. Review and comment: each DST has a free text space for providing comments of any kind that can contribute to the improvement of the tool or to improve the description within the database.

4. Search engine: two search options are available: free text search by the user’s own selected keywords, and search by (fixed) categories.

### Populating the database and controlling the quality of entries

In order to identify the available DSTs, we have formulated a procedure that allowed for both information gathering and content control. These steps were followed:

1. Identify available DSTs through the HENVINET partner network and through literature review by the DST team.

2. Contact DSTs provider or user to collect initial DSTs information in a standard initial contact form.

3. Identify ‘tutors’, or experts providing initial information, to compile and upload the database entries.

4. Identify ‘supervisors’, or experts knowledgeable on the DST subject, to review and complete the DSTs information, to authorise release of the DSTs to the public.

5. Undertake coverage assessment of the DSTs using predefined criteria, and provide this information online.

The role of the 'tutors' is solely to review the available information on the DST and enter it into the database. The role of the 'supervisors' is to review the entries, and to assess the DST regarding validation and current application. In this process, both the “tutors” and the “supervisors” are independent of the DST owner/provider.

Access to changes in the MDB is differentiated: read and search access is public, adding records require registration; a registered user can change the entry they have made. During the project (end April 2010), the 'tutors' and the 'supervisors' were authorized to change records. The owners of the DST, as far as they were known, were notified about the entry.

### Information gathering on existing decision support tools – initial contact

For initial contact, an entry form (see Additional file 2) was designed and distributed to either DST providers or DST users, or even simply to people with potential information about a DST. This initial contact form consisted of two parts, namely the contact person information and DST information. The first part included details on the contact person and the person’s organization, whereas the second part included details on a DST such as its title, category, web link and a short description. A total of 34 completed forms were received through direct contacts.

In addition to the directly identified DSTs, a literature review and an online search were undertaken to identify further DSTs. For each DST, a HENVINET partner completed the contact form. 76 additional DSTs were identified through this process, a total of 110 DSTs with brief descriptions as a basis for further work.

### Uploading information

The contact persons were asked to upload full DST information to the DST entry template. In a few cases, the initial contact person did not wish to upload the information due to a limited knowledge of the details, and was replaced by a more expert colleague, either internal or external to the HENVINET partnership. A total of 78 DSTs were uploaded into an online MDB.

### Quality control of entries

After uploading DSTs, the information was reviewed. Each DST was assigned to a HENVINET partner with experience in the sector (a ‘supervisor’). The review also included an evaluation of the DSTs regarding their use. The following six evaluation criteria were applied: 1) user friendliness (how easy is it for the user to use the DST?); 2) causal chain approach (how does the DST relate to the causal chain?); 3) robustness (how reliable is the DST?); 4) user application history (how often has the DST been used and by whom?); 5) applicability (how widely can the DST be applied?), and 6) uncertainty (has the DST been given a thorough review with regard to uncertainty?). The assessment of the DSTs was conducted in simple manner with three categories for each criterion. After the review and evaluation, the ‘supervisors’ had right to publish the contents in the MDB.

### Assessment of the coverage

To help identify any gaps in coverage of DSTs and as a basis for recommendations for further research and development of DSTs, we have summarized the database entries. The following six categories of DSTs are recognized: database, guideline, handbook, indicator, methodology and software model [12].

## Results

Contact information is available for 110 DSTs. After ‘tutors’ uploaded and ‘supervisors’ reviewed the entries, a web-based MDB with 67 DSTs (Additional file 3) is accessible through the HENVINET networking portal http://www.henvinet.eu and the HENVINET project website http://henvinet.nilu.no.

**Figure 1:**
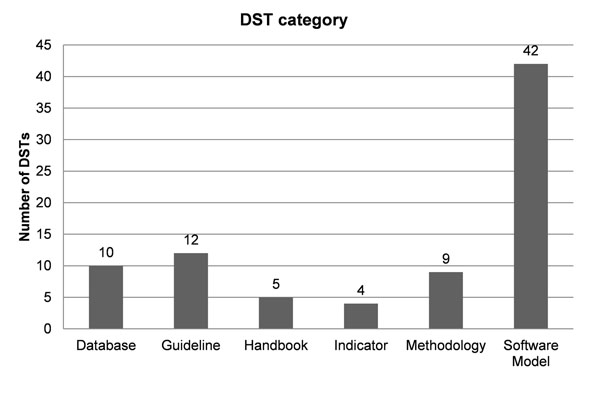
Overview of the different DSTs categories.

**Figure 2:**
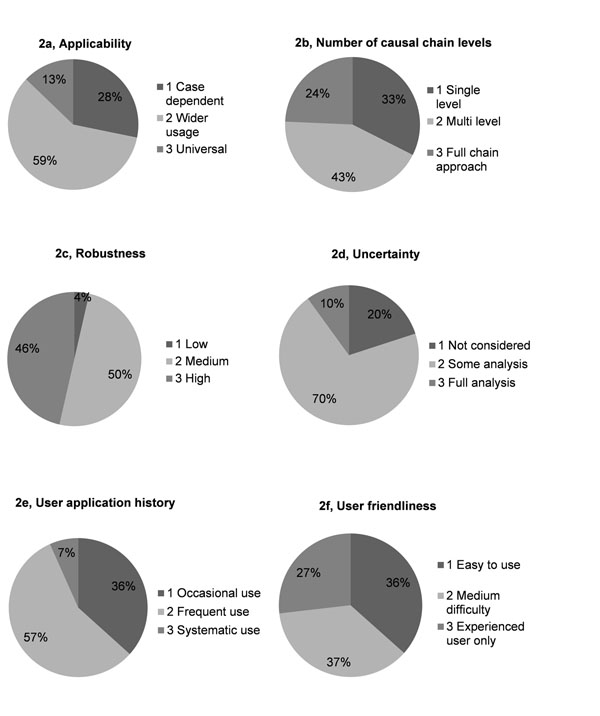
Evaluation of DSTs.

**Figure 3:**
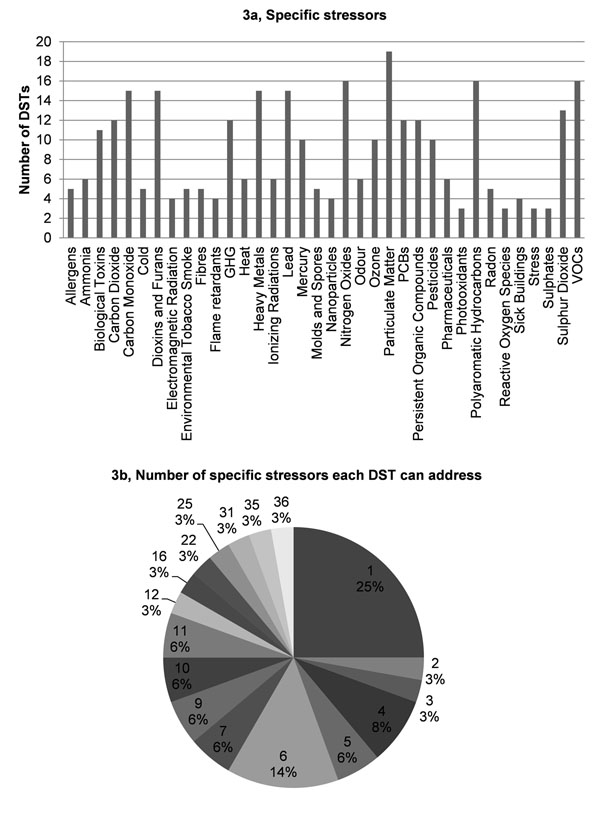
Specific stressors addressed by DSTs.

**Figure 4:**
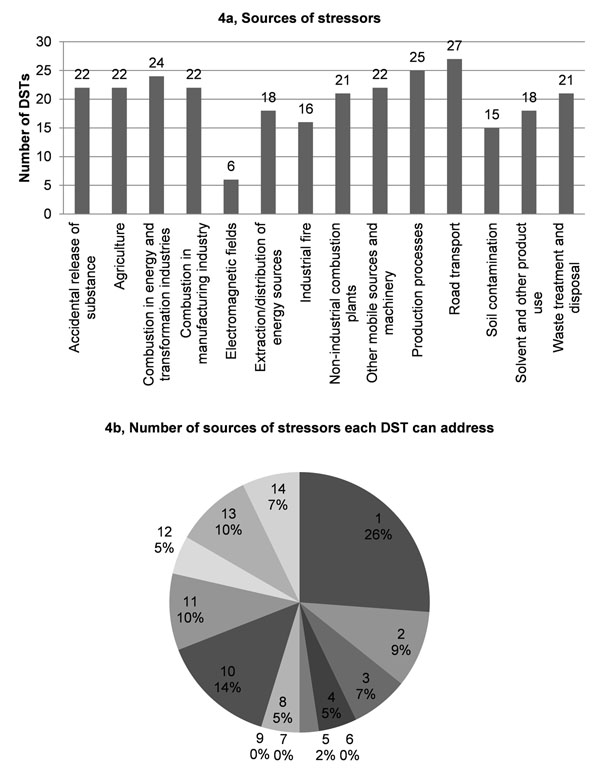
Sources of stressors addressed by DSTs.

**Figure 5:**
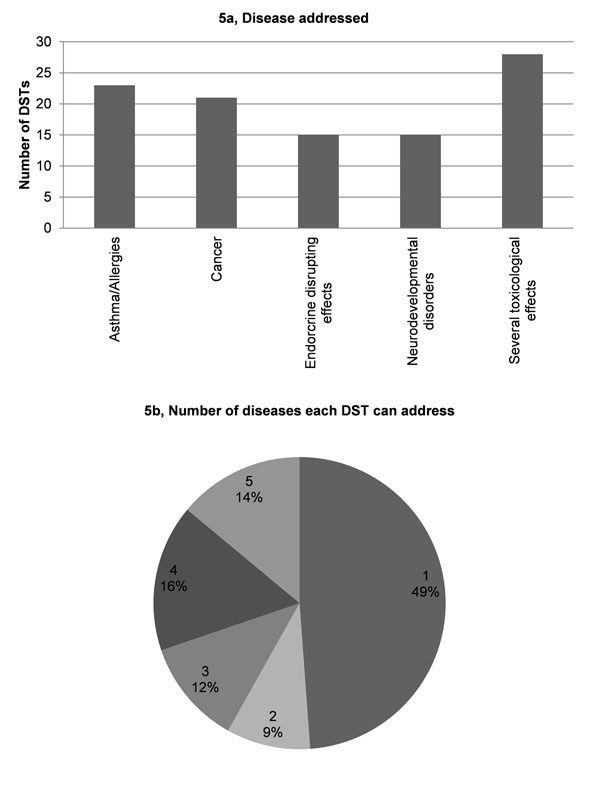
Diseases addressed by DSTs.

**Figure 6:**
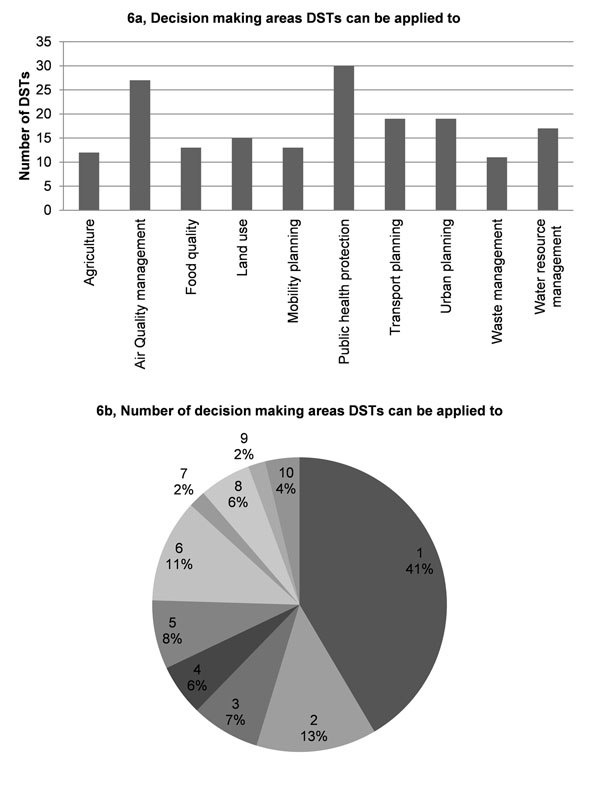
Decision making areas for DSTs.

Categorization results showed that the majority of the DSTs are software models (Fig. 1). Most DSTs 1) are for wider use (Fig. 2a); 2) are multi-level (Fig. 2b); 3) show either medium or high robustness (Fig. 2c); 4) provide some analysis on uncertainty (Fig. 2d); 5) are characterized by frequent use (Fig. 2e); 6) are about equally divided between two levels, i.e., easy to use (37%) and medium difficulty to use (36%) (Fig. 2f).

Validation results showed that 1) most DSTs are designed to address the most common pollutants found in the atmosphere, e.g., PM, NOx, VOCs and poly-aromatic hydrocarbons (Fig. 3a); 2) 25% of the DSTs address only one environmental stressor. The majority of DSTs (52%) are relevant for 4 to 11 stressors, whereas 3% are relevant for all the specified 36 stressors (Figs. 3a, 3b); 3) most DSTs address road transport, followed closely by industrial production processes and combustion in energy and transformation industries (Fig. 4a); 4) more than 25% of the DSTs address only one pollution source. A large proportion of DSTs (42%) cover from 1 to 3 sources, whereas another large proportion (46%) cover 10 or more stressors (Fig. 4b); 5) the four priority issues - asthma and allergies, cancer, neurodevelopment disorders and endocrine disrupting effects - are quite evenly addressed (15-23%) by the DSTs (Fig. 5a). Slightly more DSTs (28%) cover the topic of toxicology; 6) almost 50% of the DSTs cover only one disease or issue (Fig. 5b).

**Figure 7:**
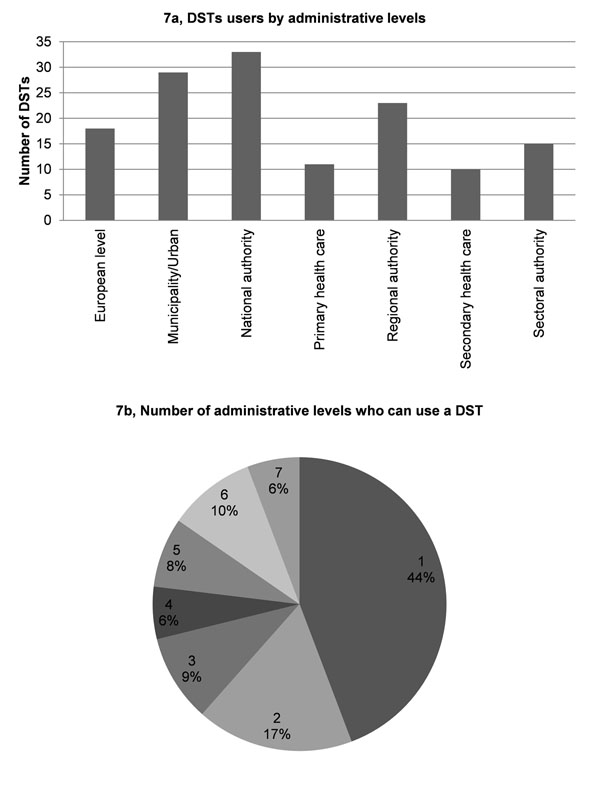
The intended administrative levels users for DST.

**Figure 8:**
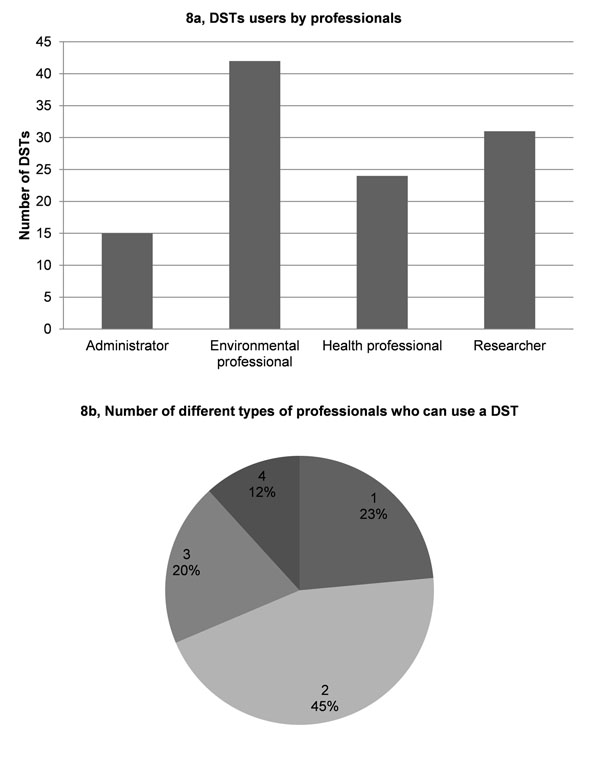
The intended professional users for DSTs.

Application results showed that 1) most DSTs are designed to address the most common decision making areas in environment and health, namely public health protection and air quality management, whereas the least addressed areas are agriculture and waste management (Fig. 6a); 2) 41% of the DSTs cover only one decision making area, whereas 86% of the DSTs cover from 1 to 6 areas (Fig. 6b); 3) the most frequent user is the national level authority (Fig. 7a); 4) 60% of the DSTs cover one or two decision-making levels (Fig. 7b). Most of DSTs combine either a single or two neighbouring levels, e.g., regional and national authority levels; 5) most DSTs are developed for use by environment professionals (Fig. 8a); 6) almost half of the DSTs can be used by professionals in two areas, whereas only 12% of DSTs can be used by all professionals. About 20% of DSTs may be used either by professionals in one or three areas (Fig. 8b). It is important to note that DSTs that can be used by professionals in two areas usually refer to the environmental professional combined with a professional from another of the three remaining areas, whereas a combination of administrator and researcher rarely occurs.

## Discussion

A number of DST repositories exist on-line, such as those by the US EPA (Environmental Protection Agency); however they usually target narrower communities, and do not offer the kind of access to information that would promote the “full chain” or DPSEEA thinking.

The HENVINET DST MDB can constantly be updated with information on additional DSTs. It provides easy access to information, is easy to manage, and it allows the user to browse data on identified DSTs, to input data on a new DST, to update the information, correct errors, or search for DSTs with specific characteristics. The MDB in particular permits the description of the purpose of the DST, its application areas, the expected users, the considered stressors and health outcomes. Where available, it provides information about how the DST was validated.

Similar review and classification of DSTs have been carried out in other fields. For instance, McLellan et al [13] have reviewed tools and methodologies “used for incorporating sustainability considerations in the design of mineral processing operations”. They note that a systematic approach is lacking, and while their framework is to support a specific industry (having in mind specific industrial process); it does have an element of the DPSIR (Driving Force–Pressure–State–Impact–Response) framework/DPSEEA in the element “understanding the effect of design choices [13]”. Hamouda et al [14], having reviewed decision support systems for water and wastewater treatment processes point out that there is a need to develop integrated systems that consider a system analysis approach. Chang et al [15] analyzed systems for solid waste management, looking at systems engineering models (e.g., cost benefit analysis, forecasting analysis), systems analysis platform (e.g., decision support tools, expert system), and assessment tools (e.g., scenario development or environmental impact assessment). Their work provides a possible framework to build on, for which there was no capacity in the current project. From this point of view, HENVINET has undertaken the first step in classifying DSTs in the area of health and environment.

From the point of view of supporting system analysis, many of the existing DSTs are classified as ‘software models’, with a majority having a ‘single type’ characteristic (e.g., one environmental stressor, one type of disease). Only a few have a more universal character. In addressing the natural complexity of E&H issues and using the most suitable methodology, there is a need to define or identify a universal framework encompassing this variety of tools. As a preparation for such activity, the achievements of existing health impact assessment (HIA) frameworks, e.g., the HIA framework defined by WHO, should be investigated. Currently, more general methodologies are being developed, amongst them is the toolbox on integrated environmental health impact assessment system (http://www.integrated-assessment.eu) developed by EU FP6 projects HEIMTSA (Health and Environment Integrated Methodology and Toolbox for Scenario Assessment) (http://www.heimtsa.eu) and INTARESE (Integrated Assessment of Health Risks of Environmental Stressors in Europe) (http://www.intarese.org), as well as an interactive wiki-based platform for communication - the Open Assessment Network (http://www.opasnet.org), which is mainly supporting open environmental assessments.

The present evaluation of E&H DSTs aimed to provide an overall and general idea of the quality and usability of the tools, based on six aspects (applicability, causal chain approach, robustness, uncertainty, user application history and user friendliness, Fig.2). This choice of aspects represents a compromise between the academic-scientific approach and the expected difficulties for users to reply with high confidence and credibility to the questions included in the online MDB.

In terms of the DST validation, the most striking points emerging from the analysis are: 1) only 3% of DTSs claim to deal with all 36 stressors; while 2) 25% of DSTs deal with only one stressor; and 3) 50% of DSTs deal with only one disease/issue. This leads to the question whether it is feasible or useful to stimulate the creation of DSTs covering more stressors and more diseases/issues. Such an integrative trend seems desirable. In designing a policy, it is essential to know the impacts on a given aspect e.g., of local importance, but limiting ourselves to a single issue, albeit perceived as the most important one at a given time, will lead to unbalanced decisions and possible long-term harm. The goal of a ‘multi diseases tools’ is evident, as is the need for ‘all stressors based’ DSTs. How can we assess environmental health if we do not consider the known or suspected stressors and effects? The development of methodologies and software tools covering increasing numbers of environmental stressors and health end points should be pursued, notwithstanding the inevitable difficulties that this implies.

Half of the DSTs are applicable for only one decision making area. What is the effect of this? We have identified 10 decision making areas (Fig. 6a), and 50% of DSTs cover only 1 of them. This is a consequence of the high number of DSTs covering only few stressors and dealing with only few diseases.

Regarding the administrative levels and the type of users using E&H DSTs, we noticed a remarkable dominance of environmental scientists and researchers compared to administrators and health professionals using DSTs. As a consequence, there is a need to develop DSTs for a wider application context, relevant to more decision making areas, and in particular, suitable for use by administrators and health professionals.

The project has identified 110 DSTs, but despite considerable effort, has managed to get structured information on only 60% of those. Better recognition of the need to identify the multiplicity of the tools, and their wider review, seems to be necessary.

## Conclusions

We have developed a common framework for DSTs in the E&H field that allows for “issues” or “systems” thinking rather than “discipline” thinking, and we have started information gathering, classification and evaluation. We have delivered a product – an operational web-based searchable DSTs MDB. The framework for DST information gathering is general, and in our opinion can cover many more areas of use and application of DSTs. The categorisation, evaluation and application descriptors are a workable compromise of our ideas of what is useful for the user to know, in order to choose an appropriate DST.

It has not been the aim to formally validate each individual DST. This is the task of any responsible DST provider, who should document their tools in a manner that would provide the user with confidence in the product.

Different DSTs that are relevant to any single disease/issue may have different inputs. This indicates that they use different determinants to achieve the same outcome, and are based on different partial understanding of underlying mechanisms. Since recommended actions are directed at changing/reducing the determinants, different DSTs will provide different advice to address the same disease/issue. Therefore there is a bias resulting from the uneven availability of information, favouring information that is readily available over perhaps more relevant but not so easily available information. This stresses the importance of the initiatives at both European and global levels that aim to secure comparable information on DSTs in the area of health and environment, and for more research on the issues linking environment and health. It also underlines the need to maximise thorough and systematic documentation of existing DSTs, using common criteria. This current work is one step on the way.

## Competing interests

The authors declare that they have no competing interests.

## Authors' contributions

HYL and AB planed this work. HYL wrote the manuscript, revised data analysis. All authors contributed to the concept development and implementation. PN, CH, EN, HYL and AY were in charge of the implementation and data gathering. MJK was in charge of the design of the technical solutions, MJK and AY were responsible for technical implementations and support. PN and CH did data analysis. AB thoroughly revised the manuscript. EN was responsible in the project for the DST work, AB was the project coordinator. All authors approved the final version.

## Supplementary Material

Additional file 1 Questionnaire for information gathering on decision support tools Click here for file

Additional file 2 Contact person information and decision support tools information Click here for file

Additional file 3 Overview of 67 DSTs with their name, category, contact person, location, and web link (--- means no available information on contact person, location or web link). *Click here for file*
