# Supplementary material for: Facilitating knowledge transfer: decision support tools in environment and health
Source: Environ Health. 2012 Jun 28;11(Suppl 1):S17. doi: 10.1186/1476-069X-11-S1-S17 (PMC3388451; doi:10.1186/1476-069X-11-S1-S17)
Supplement: Additional file 1 — Questionnaire for information gathering on decision support tools [file 1476-069X-11-S1-S17-S1.docx]

## Additional file 1 Questionnaire for information gathering on decision support tools

| **Field** | **Comment/attributes** |
| --- | --- |
| DST category | methodology  software tool  handbook  indicator  guideline  database  other |
| Thematic area (reference to the causal diagram) | sources of environmental stressors  dispersion processes and pathways  exposure of population  population behaviour  health endpoints  overall evaluation  other |
| Environmental pathways considered by the tool | noise  air quality  food  water  electromagnetic fields  other |
| Decision making area where the tool is used | public health protection  land use  urban planning  transport planning  waste management  food chain  mobility planning  air quality management  water resources management  other |
| Rings of the causal chain considered | sources  concentrations  exposure  dose  health effects  impacts |
| Risk groups | children  elderly  pregnant  ethnicity  gender  all |

## Additional file 1 Questionnaire for information on gathering decision support tools (cont)

| **Field** | **Comment/attributes** |
| --- | --- |
| administrative level at which the DST results are mainly used | municipality/urban  regional authority  sectoral authority  national authority  European level  international |
| Who is the main intended immediate operator of the tool | administrator  health professional  environmental professional  E&H researcher  citizen  other |
| Does the DST address the following environment related diseases? | asthma and allergies  cancer  neuro-developmental disorders  endocrine disrupting effects  cardiovascular diseases  other |
| Why should the tool be used? E.g. points of strength | free text |
| Does the DST takes into account uncertainty? If yes in which way? | free text |
| To what extent has the DST been validated? | free text |
| List of DST application/validations | free text |
| Example of validation | free text |
| Example of application | free text |
| Funding source | international  European Commission  national  self-funding |
| Software requirements | unix  linux  Windows  mac  solaris |
| User technical and scientific competencies required | free text |
| Languages | English  French  Italian  German  ...etc |
| DST detailed description | file to be uploaded |
