# Supplementary material for: Facilitating knowledge transfer: decision support tools in environment and health
Source: Environ Health. 2012 Jun 28;11(Suppl 1):S17. doi: 10.1186/1476-069X-11-S1-S17 (PMC3388451; doi:10.1186/1476-069X-11-S1-S17)
Supplement: Additional file 2 — Contact person information and decision support tools information [file 1476-069X-11-S1-S17-S2.docx]

## Additional file 2 Contact person information and decision support tools information

| **No.** | **Contact person information and DSTs information** |
| --- | --- |
| 1 | Contact person title |
| 2 | Contact person first name |
| 3 | Contact person last name |
| 4 | Contact person organization short name |
| 5 | Contact person organization full name |
| 6 | Web link to contact person organization |
| 7 | Address |
| 8 | City |
| 9 | Country |
| 10 | Contact person E-mail |
| 11 | Contact person telephone |
| 12 | Role of the contact person (DST provider, DST user or person informed on the DST) |
| 13 | DST name |
| 14 | DST category (choose one of database, software model, methodology, indicator, handbook, guideline) |
| 15 | Short description of DST (max 1000 characters) |
| 16 | Web link or other reference of the DST |
