# Supplementary material for: Facilitating knowledge transfer: decision support tools in environment and health
Source: Environ Health. 2012 Jun 28;11(Suppl 1):S17. doi: 10.1186/1476-069X-11-S1-S17 (PMC3388451; doi:10.1186/1476-069X-11-S1-S17)
Supplement: Additional file 3 — Overview of 67 DSTs with their name, category, contact person, location, and web link (--- means no available information on contact person, location or web link). [file 1476-069X-11-S1-S17-S3.docx]

## Additional file 3 Overview of 67 DSTs with their name, category, contact person, location, and web link (--- means no available information on contact person, location or web link).

| **DST name** | **Category** | **Contact person** | **Location** | **Web link** |
| --- | --- | --- | --- | --- |
| Acid Mine Drainage-AMDTreat | Software model | Nikmo, Juha | Europe | <http://www.fmi.fi/research_air/air_55.html> |
| Action plan | Methodology | Hans Keune | Europe | [http://www3.interscience.wiley.com/journal/122222075/abstract](http://www3.interscience.wiley.com/journal/122222075/abstract%20) |
| AirGIS-a GIS-based modeling system for estimation of traffic-related air quality and human exposure | Software model | Steen Solvang Jensen | Asia, Denmark, Europe, North-America | [http://airgis.dmu.dk](http://airgis.dmu.dk/) |
| AirQUIS-Air Quality Management Information System | Software model | Rune Ødegård | Global | [http://www.airquis.com](http://www.airquis.com/) |
| AirWare | Software model | Desk Opsis | Global | http://www.ess.co.at/AIRWARE |
| ARAMS - Adaptive Risk Assessment Modeling System | Software model | Brooke Magnanti | North-America | [http://el.erdc.usace.army.mil/arams](http://el.erdc.usace.army.mil/arams%20) |
| Assessment Follow-Up (FU) at 5 years of age | Guideline | --- | Europe | --- |
| Benchmark Dose Software (BMDS) | Software model | --- | North-America | <http://www.epa.gov/ncea/bmds/about.html> |
| BENMAP-Environmental Benefits Mapping and Analysis Program | Software model | --- | North-America | <http://www.epa.gov/air/benmap> |
| BIOCHLOR | Software model | Brooke Magnanti | North-America | <http://www.epa.gov/ada/csmos/models/biochlor.html> |
| BIOPLUME III | Software model | Brooke Magnanti | Global | <http://www.epa.gov/ahaazvuc/csmos/models/bioplume3.html> |
| CAMX | Software model | --- | --- | [http://forecast.uoa.gr](http://forecast.uoa.gr/) |
| Calendex | Software model | Brooke Magnanti | North-America | [http://www.exponent.com/calendex_software](http://www.exponent.com/calendex_software%20) |
| Children's Environmental Health Profile in Argentina | Database, guideline, indicator | --- | --- | http://www.aamma.org/wp-content/uploads/2009/05/profile_of_children_environmental_health_in_argentina_sanaprofile_-englishversion.pdf |
| City of Helsinki Readiness Plan for air Quality Episodes | Guideline | --- | Finland | http://http//www.hel.fi/wps/wcm/connect/f47445804a17230ba178e93d8d1d4668/Julkaisu_10_07_net.pdf?MOD=AJPERES&CACHEID=f47445804a17230ba178e93d8d1d4668 |
| Computer-Aided Management of Emergency Operations | Database | --- | North-America | <http://www.epa.gov/oem/content/cameo/what.htm> |
| Consumer Exposure | Methodology | Peter van den Hazel | Global | <http://www.rivm.nl/en/healthanddisease/productsafety/ConsExpo.jsp> |
| COSIMO-Cost of compliance simulation model | Software model | Pierre Gerber | Asia | --- |
| Definition Small for Gestational Age and Intra-Uterine Growth Retardation | Guideline | --- | Global | [HENVINET-DST-CONTACTS-FORM-definitions of SGA and IUGR .doc](http://www4.nilu.no/applications/farcry_henvinet/www/metadatabase/index.cfm?fuseaction=view.download&identifier=%7b116A906B-17A4-8DB2-8C5C-4198867AD03A%7d) |
| E-FAST Exposure and Fate Assessment Screening Tool | Software model | --- | United States | <http://www.epa.gov/oppt/exposure/pubs/efast.htm> |
| Effect of Nanoparticles on Environment & Health: Causal diagram | Database, methodology, software model | --- | Global | http://henvinet.nilu.no/EvaluationofKnowledge/tabid/1333/language/en-US/Default.aspx |

## Additional file 3 Overview of 67 DSTs with their name, category, contact person, location, and web link (--- means no available information on contact person, location or web link) (cont).

| **DST name** | **Category** | **Contact person** | **Location** | **Web link** |
| --- | --- | --- | --- | --- |
| EnviMan | Database, software model | Desk Opsis | Global | [http://www.opsis-gmbh.de/monitoring/enviman.asp#](http://www.opsis-gmbh.de/monitoring/enviman.asp) |
| European Union system for the evaluation of substances | Software model |  | Europe | <http://www.rivm.nl/rvs/overige/risbeoor/Modellen/EUSES_Support.jsp> |
| ESCAPE-Expert System for Consequence Analysis using a PErsonal computer | Software model | Juha Nikmo | Europe | <http://www.fmi.fi/research_air/air_55.html> |
| EXPosure to Air pollution, especially to Nitrogen Dioxide and particulate matter | Software model | Anu Kousa | Finland | [http://http://www.aka.fi/sytty/abstracts/kukko.htm](http://http/www.aka.fi/sytty/abstracts/kukko.htm) |
| F/S PLUS | Software model | --- | United States | <http://www.epa.gov/region5fields/htm/software.htm> |
| FIELDS - FIeld EnvironmentaL Decision Support | Software model | --- | United States | [http://www.epa.gov/region5fields](http://www.epa.gov/region5fields%20) |
| Finish national air quality portal | Database | Virpi Tarvainen | Finland | <http://www.fmi.fi/en/index.html> |
| Framework for Decision Making in the Field of Environment and Health | Methodology | Peter van den Hazel | Europe | <http://www.rivm.nl/bibliotheek/rapporten/609026003.pdf> |
| GeoSEM | Software model | --- | --- | <http://esc.syrres.com/geosem/default.htm> |
| Global Burden of Disease measured as DALY | Handbook | --- | Global | [http://www.who.int/topics/global_burden_of_disease/en](http://www.who.int/topics/global_burden_of_disease/en%20) |
| Health Effect Screening | Guideline, methodology | Peter van den Hazel | Europe | [http://www.vrom.nl/get.asp?file=docs/publicaties/w309.pdf&dn=w309&b=vrom n](http://www.vrom.nl/get.asp?file=docs/publicaties/w309.pdf&dn=w309&b=vrom%20n) |
| Health Impact Assessment | Guideline, handbook, methodology | --- | Global | [http://www.who.int/hia/en](http://www.who.int/hia/enhttp://en.wikipedia.org/wiki/Health_Impact_Assessment#Overview ) |
| Hybrid Single Particle Integrated Trajectory Model | Software model | Roland Draxler | Global | --- |
| Hydrocarbon Spill Screening Model | Software model | --- | United States | <http://www.epa.gov/ada/csmos/models/hssmwin.html#Installation> |
| IEUBK-integrated exposure uptake biokinetic model | Software model | Brooke Magnanti | North-America | <http://www.epa.gov/superfund/lead/products.htm> |
| INDEMIAP | Software model | --- | Europe | --- |
| INTARESE/HEIMTSA toolbox | Database | Fintan Hurley | Europe | http://www.integrated-assessment.eu |
| Integrated Computational Assessment of Air Quality via Remote Observations Network | Database, software model | Denis Sarigiannis | Global | http://icaros-net.jrc.ec.europa.eu |
| Integrated Risk Information System | Software model | --- | North-America | <http://cfpub.epa.gov/ncea/iris/index.cfm> |
| ISHTAR - Integrated Software for Health, Transport efficiency and Artistic heritage Recovery | Software model | Emanuele Negrenti | Europe | [ISHTAR for EUROCITIES - Utrecht Sept 2007.ppt](http://www4.nilu.no/applications/farcry_henvinet/www/metadatabase/index.cfm?fuseaction=view.download&identifier=%7b5682B046-17A4-8DB2-8C43-294E52BB4CD9%7d) |
| J&E Model - Johnson and Ettinger Model for Subsurface Vapor Intrusion into Buildings | Software model | --- | United States | <http://www.epa.gov/oswer/riskassessment/airmodel/johnson_ettinger.htm> |
| Marine antifoulant model to predict environmental concentrations (MAMPEC) | Software model | Bert van Hattum | Global | http://delftsoftware.wldelft.nl/index.php?option=com_docman&task=cat_view&gid=81&dir=DESC&order=hits&limit=5&limitstart=5 |
| Monitoring and Remediation Optimization System Software (MAROS) | Software model | Mindy Vanderford | --- | http://www.gsi-net.com/software/free-software/maros.html |

## Additional file 3 Overview of 67 DSTs with their name, category, contact person, location, and web link (--- means no available information on contact person, location or web link) (cont).

| **DST name** | **Category** | **Contact person** | **Location** | **Web link** |
| --- | --- | --- | --- | --- |
| OECD Test Guideline 426 | Guideline | --- | Global | [http://dx.doi.org/ doi:10.1289/ehp.11447](http://dx.doi.org/%20doi:10.1289/ehp.11447) |
| Opasnet | Database, guideline, handbook, indicator, methodology, software model | Jouni Tuomisto | Global | [http://en.opasnet.org](http://en.opasnet.org/) |
| Optimized expert system for conducting environmental assessment urban road traffic | Software model | Sokhi, Ranjeet | Europe | http://www.eu-oscar.org |
| Physiologically Based PharmacoKinetic (PBPK) model | Software model | --- | Europe | <http://www.hsl.gov.uk/capabilities/pbpk.htm> |
| Platform for Exposure Assessment | Database, guideline | Arja Asikainen | Global | <http://www.ktl.fi/expoplatform> |
| Practical guidance for assessment of disease burden at national and local levels | Guideline | --- | Global | [http://www.who.int/quantifying_ehimpacts/en](http://www.who.int/quantifying_ehimpacts/en%20) |
| Practical guide to involve stakeholders in the WFD process | Handbook | Jaap Van der Vlies | Global | <http://www.socopse.se/decisionsupportsystem/tools/stakeholderinvolvement.4.3d9ff17111f6fef70e9800053344.html> |
| Preconception Counseling | Guideline | --- | Global | --- |
| QWASI | Software model | Ruud Baartmans | Global | <http://www.trentu.ca/academic/aminss/envmodel/models/QWASI310.html> |
| RAINS/GAINS ITALY | Software model | Tiziano Pignatelli | Italy | [http://http://minni.bologna.enea.it:8080/minni/sistema/rains-gains](http://http/minni.bologna.enea.it:8080/minni/sistema/rains-gains) |
| SKIRON/Dust | Software model | Pierre Gerber | Asia | [http://forecast.uoa.gr](http://forecast.uoa.gr/) |
| SOCOPSE DSS-SOCOPSE Decision Support System | Handbook | Ruud Baartmans | Global | <http://www.socopse.se/decisionsupportsystem> |
| SourceDK-Remediation Timeframe Decision Support System | Software model | Shahla Farhat | Global | <http://www.frtr.gov/decisionsupport/DSTMatrix.htm> |
| STEDOM | Software model | Victoria Aleksandropoulou |  | --- |
| Supplying Sustainable Agriculture Production | Indicator, software model | Carlo Riparbelli | Italy | http://susapnetwork.iambientale.it |
| Supplying Sustainable Agriculture Production Web | Database, indicator | Carlo Riparbelli | Italy | http://susapnetwork.iambientale.it |
| STRAW-Support for the Treatment and Recycling of Animal Waste | Software model | Pierre Gerber | Asia | --- |
| ENHIS-The European Environment and Health Information System | Database | Michal Krzyzanowski | Europe | http://www.enhis.org |
| DALYs-The Uniform Disability Adjusted Life Years | Methodology | --- | Europe | <http://www.who.int/entity/water_sanitation_health/dwq/rivmrep.pdf> |
| Traffic Emission and Energetics | Software model | Emanuele Negrenti | Europe, Oceania | [TEE in Prague 2006.ppt](http://www4.nilu.no/applications/farcry_henvinet/www/metadatabase/index.cfm?fuseaction=view.download&identifier=%7bED77554E-17A4-8DB2-8CFA-29CC24AFDD2B%7d) |
| TRANsport Direct Impacts | Software model | Emanuele Negrenti | Europe | --- |
| UDM-FMI, CAR-FMI and EXPAND | Software model | Mia Pohjola | Europe | --- |
| URBIS | Software model | Ernst Meijer | Europe | [www.tno.nl/urbis](http://www.tno.nl/urbis) |
